# Estimation of Time-Course Core Temperature and Water Loss in Realistic Adult and Child Models with Urban Micrometeorology Prediction

**DOI:** 10.3390/ijerph16245097

**Published:** 2019-12-13

**Authors:** Toshiki Kamiya, Ryo Onishi, Sachiko Kodera, Akimasa Hirata

**Affiliations:** 1 Department of Electrical and Mechanical Engineering, Nagoya Institute of Technology, Nagoya 466-8555, Japan; t.kamiya.346@nitech.jp (T.K.); kodera@nitech.ac.jp (S.K.); 2 Center for Earth Information Science and Technology, Japan Agency for Marine-Earth Science and Technology, Yokohama 236-0001, Japan; onishi.ryo@jamstec.go.jp; 3 Center of Biomedical Physics and Information Technology, Nagoya Institute of Technology, Nagoya 466-8555, Japan; 4 Frontier Research Institute of Information Science, Nagoya Institute of Technology, Nagoya 466-8555, Japan

**Keywords:** large-eddy simulation, microclimate and micrometeorology, risk management, thermal stress, thermophysiology, urban environment

## Abstract

Ambient conditions may change rapidly and notably over time in urban areas. Conventional indices, such as the heat index and wet bulb globe temperature, are useful only in stationary ambient conditions. To estimate the risks of heat-related illness, human thermophysiological responses should be followed for ambient conditions in the time domain. We develop a computational method for estimating the time course of core temperature and water loss by combining micrometeorology and human thermal response. We firstly utilize an urban micrometeorology prediction to reproduce the environment surrounding walkers. The temperature elevations and sweating in a standard adult and child are then estimated for meteorological conditions. With the integrated computational method, we estimate the body temperature and thermophysiological responses for an adult and child walking along a street with two routes (sunny and shaded) in Tokyo on 7 August 2015. The difference in the core temperature elevation in the adult between the two routes was 0.11 °C, suggesting the necessity for a micrometeorology simulation. The differences in the computed body core temperatures and water loss of the adult and child were notable, and were mainly characterized by the surface area-to-mass ratio. The computational techniques will be useful for the selection of actions to manage the risk of heat-related illness and for thermal comfort.

## 1. Introduction

With the advance of global warming, heat-related morbidity and mortality has become a serious social issue worldwide. In Japan, the number of ambulance dispatches related to heat-related illness has remained at over 40,000 since 2012, and reached 90,000 in 2018 [1]. The patients with heat-related illnesses were mainly from urban areas (near the Tokyo, Osaka and Aichi prefectures), which is partly attributable to the large population of those areas. In addition to global warming, the “urban heat island effect” cannot be neglected. According to the World Population Prospects (2017) of the United Nations (UN), two-thirds of the population will live in urban cities (urban population: 6.7 billion; total population: 9.8 billion) by 2050 [2]. As such, heat mitigation in urban areas is essential.

The wet-bulb temperature (WBGT), as defined by the International Organization for Standardization (ISO), is often used as a metric for thermal stress, especially in occupational safety [3]. WBGT is also used as a metric in municipalities. In addition, heatstroke warning systems have been introduced using several indices for thermal stress in many countries [4]. Conventional indices are mainly based upon the measurement every several hours with an instrument fixed in the shade in each municipality. However, in our daily life, the ambient condition may change rapidly with time and sharply in space, in addition to other conditions including the material of the ground and the presence of street trees and buildings in urban areas.

Even in the same environment, the risk of heat-related illness varies among population, i.e., children, adults and elderlies. Well-known physiological parameters linked to heat-related illnesses include core temperature elevation and water loss. The American Conference of Government Industrial Hygienists (ACGIH) standard recommended the prevention of core temperature elevation by less than 1 °C and empirical guidelines for water intake for healthy workers [5]. It would be useful for public enlightenment regarding heat-related illness if information, based on the estimation of core temperature and water loss, could be provided to the general public with high accuracy. Thermal comfort, or the risk management of heat-related illness, are the concerns about health conditions due to expected climate changes in future [6]. Therefore, it is required to develop a computational assessment of core temperature elevation and water loss considering the realistic ambient environment.

There are primarily two aspects to be considered for this purpose. The first aspect is the estimation of the microscale environment. Real-time operational urban micrometeorology simulations are needed, with a fine spatial resolution (on the order of several meters) utilizing “Internet of Things” (IoT) sensor data. The sensor data, in turn, are strongly influenced by human activity, buildings etc. [7,8]. Such simulations would facilitate real-time heat mitigation for individuals and urban drone logistics. In that regard, such simulations have started to be applied for the mitigation of urban heat islands, and also for predicting the impacts of heat mitigation strategies on a pedestrian environment [9,10].

The other aspect is an appropriate estimation of core temperature and water loss for different generations. Children are known to be vulnerable to the heat, because of their larger body-surface-area-to-mass ratio [11]. Thermoregulatory models are becoming significantly useful to predict thermal stress [12,13,14]. Only a few studies have evaluated the thermal stress in different generations, and even then, only with fixed conditions [15,16].

We developed the estimation techniques of micrometeorology and demonstrated their validity by comparing with the observation data in our previous study [17]. It has been confirmed that the simulation can reproduce the statistical variance in urban street scales, while the reliability of average statistics depends upon the boundary conditions, which are given by meteorological simulations. The human thermophysiological responses have also been modeled considering dependencies such as age and region, and have been validated by comparing with the volunteer study in [16,18,19,20].

In this study, we develop an integrated computational method between the microscale environment and human thermoregulation simulations in models of an adult and child, with an eye toward realistic risk estimation and management. Specifically, resultant information can be useful for mitigation strategies and the risk management of heat-related illness; how often and how much water the people should take. Furthermore, the estimation of the core temperature and water loss could be coupled with a multi-agent model to account for individual behavior and to assist in the selection of measures to manage the risk of heat-related illness.

## 2. Building-Resolving Urban Micrometeorology Simulation 

A multiscale coupled atmospheric–oceanic model named the “Multi-Scale Simulator for the Geoenvironment” (MSSG), developed at the Japan Agency for Marine–Earth Science and Technology (JAMSTEC), can simulate global, intermediate (meso), and urban scales [17,21,22,23]. We use the atmospheric module of the MSSG (i.e., MSSG-A), which can be considered as a building-resolving large-eddy simulation (LES) model coupled with a three-dimensional radiative transfer model [17]. The governing equations for MSSG-A are the transport equations for compressible flow (which consist of the conservation equations for mass, momentum and energy), and the mixing ratios of water substances, including water vapor, liquid and ice cloud particles. 

**Figure 1:**
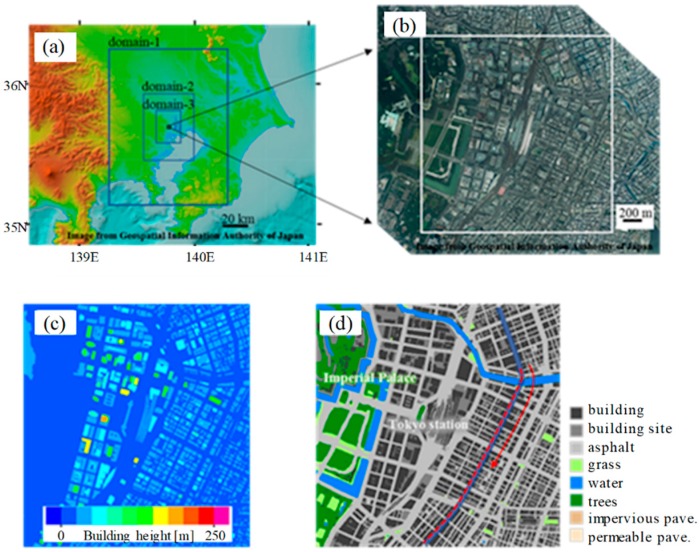
Computational domain for the urban micrometeorology prediction: (**a**) mesoscale simulation domain with three nested domains, (**b**) building-resolving large-eddy simulation (LES) domain, (**c**) building height, and (**d**) land usage in the LES domain. Red dashed line in (**d**) indicates the target route of pedestrians.

Figure 1 shows the computational domains for the present set of simulations. The mesoscale simulations adopted three two-way-coupled nested systems (Figure 1a), with horizontal resolutions of 1 km (domain 1), 300 m (domain 2) and 100 m (domain 3), centered at the urban simulation domain (Figure 1b). For all three nested systems, 55 vertical layers (lowest-layer height: 75 m) were used for the 40-km height domain. The boundary and initial conditions for the mesoscale simulations were obtained from the Japan Meteorological Agency (JMA) mesoscale analysis data.

We performed an LES for a Tokyo metropolitan built-up area, as shown in Figure 1b–d. The Tokyo domain was centered at 35.680882° N and 139.767019° E, and covered a 2 km × 2 km horizontal area with a 5-m horizontal resolution. The domain height was set to 1500 m, and 151 vertical grid points were used. The vertical grid spacing below the height of 500 m was uniformly set to 5 m, whereas the spacing above was extended continuously. For the wall boundary conditions, the friction drag at the wall surfaces was given by a logarithmic law with specified roughness parameters. We adopted the same physical schemes for the micrometeorological simulation as those described in [17]. 

A typical hot summer time in Tokyo was adopted for analysis: 14:00–15:00 on Japan Standard Time (JST) on 7 August 2015. The JMA Tokyo Observatory in Otemachi observed a maximum temperature of 36.5 °C during the hour. The results from the first 10 min time integrations were discarded, and the remaining 50 min of results were used.

**Figure 2:**
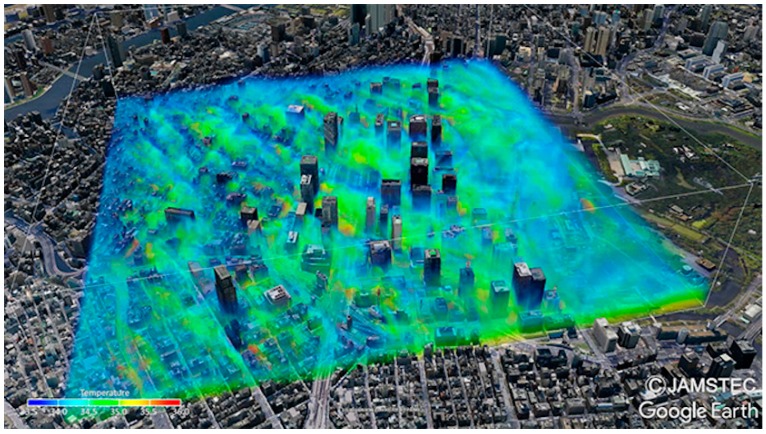
Three-dimensional (3D) distribution of instantaneous air temperature for 14:30 JST on 7 August 2015.

Figure 2 shows an example of the three-dimensional distribution of instantaneous air temperature at 14:30 JST on 7 August 2015. The “Volume Data Visualizer for Google Earth (VDVGE)” [24] was utilized for the visualization. The air was warmed up by the land surface, which was heated by solar radiation. The buoyant motion and the turbulent transportation of the warmed air formed the puffy structure of the temperature distribution.

For the three-dimensional radiative transfer process, two wavelength bands for shortwave radiation and one for longwave radiation were calculated. In addition to ambient temperature, wind velocity and relative humidity, those radiations, from which the mean radiant temperature can be calculated, were used as input parameters in the human thermoregulation computation.

## 3. Thermal and Electromagnetic Analysis in Humans

### 3.1. Anatomical Human Model

**Figure 3:**
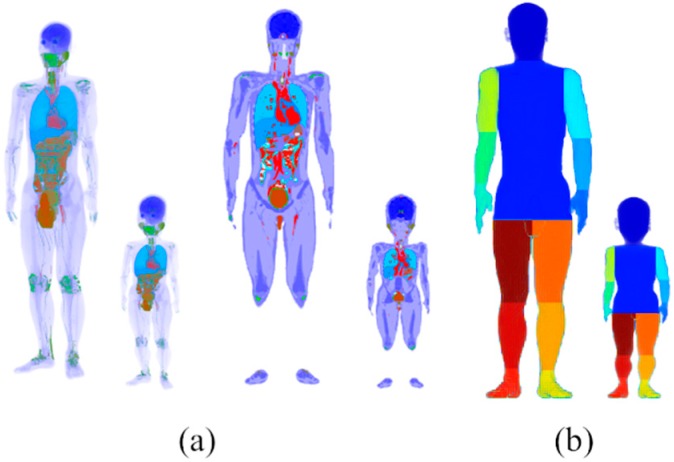
(**a**) Anatomical human adult (left) and three-year-old child models and (**b**) the definition of body parts for computing blood temperature.

A Japanese male and three-year-old child models were used as references in this study [25,26]. The height and weight of the adult model were 1.73 m and 65 kg (5′ 8″ and 143.3 lb), respectively, whereas those of the child models were 0.90 m and 13 kg (2′ 11″ and 28.67 lb), respectively. The surface areas of the adult and child models were 1.78 m^2^ and 0.56 m^2^, respectively [25,26,27]. Figure 3 illustrates the anatomical human models and the definitions of body parts for computing the blood temperature. The latter body-part models are considered to approximately represent a compartment model (e.g., cylinders as in [28]), as often used for human temperature computation. We then increased the number of body parts from five [16] to thirteen, to more accurately simulate different limb temperatures [20].

### 3.2. Thermal Analysis

A detailed explanation of our in-house computational code, developed at the Nagoya Institute of Technology, was given in our previous study [16,20]. In addition to ambient temperature, humidity and wind velocity, the heat owing to solar and infrared radiations, including reflected radiations, was also considered (see Section 3.3). The thermoregulation of the human models were those of an adult grown in a temperate zone [16,20]. The 3-year-old child is also modeled as to distinguish between arterial and venous temperatures for the first time (mentioned in Section 3.2.2). Our code was vectorized and parallelized to perform fast estimation of core temperature elevation and sweating. 

#### 3.2.1. Bioheat Equation

The temperature in the human models was followed in the time domain by solving the Pennes’ bioheat transfer equation (BHTE) [29]. A generalized bioheat equation, considering the thermoregulation and core temperature change [18], can be given as:

(1) $$C(r)\rho (r)\frac{\partial T(r,t)}{\partial t}=\nabla \cdot \left(K(r)\nabla T(r,t)\right)+M(r,t)+\sigma (r)E^{2}\left(r,t\right)-B(r,t)\left(T(r,t)-T_{B}(m,t)\right),$$

where *T* denotes the tissue temperature at position *r* and time *t*; *T_B_* denotes the blood temperature of each body part (*m* = 1, …, 13, where *m* represents the body parts shown in Figure 3b); *C* denotes the specific heat of the tissue; *ρ* denotes the tissue density; *K* denotes the thermal conductivity of the tissue; *M* denotes the metabolic heat generation; *σ* denotes the conductivity of the tissue; *E* denotes the internal electric field caused by solar radiation; and *B* denotes a term associated with blood perfusion. The metabolic heat includes the basal heat and the activity heat.

The boundary conditions between air and tissue for Equation (1) are expressed as:

(2) $$-K(r)\frac{\partial T(r,t)}{\partial n}=H(r)\cdot \left(T(r,t)-T_{a}(t)\right)+EV(r),$$

where *H*, *T_a_*, and *EV* denote the heat transfer coefficient, ambient temperature and evaporative heat loss, respectively. The heat transfer coefficient includes the convective and radiative heat losses, and varies according to the following equation [30]:

(3) $$\begin{array}{l} H\left(r,t\right)=\left(H_{C}\left(r,t\right)+H_{R}\left(r,t\right)\right)/R_{sf},\\ H_{C}\left(r,t\right)={\left[a_{nat}\cdot {\left(T_{sf}\left(t\right)-T_{e}\left(t\right)\right)}^{\frac{1}{2}}+a_{frc}\cdot v+a_{mix}\right]}^{\frac{1}{2}},\\ H_{R}\left(r,t\right)=\sigma \cdot \psi \cdot \varepsilon_{sf}.\varepsilon_{sr}\times \left[{\left(T_{sf}\left(t\right)+273\right)}^{2}+{\left(T_{a}\left(t\right)+273\right)}^{2}\right]\times \left[\left(T_{sf}\left(t\right)+273\right)+\left(T_{a}\left(t\right)+273\right)\right], \end{array}$$

where *H_c_* is the convective heat transfer coefficient; *H_R_* is the radiative heat transfer coefficient; *T_sf_* is the average body surface temperature; *v* is wind velocity; *a_na_*_t_, *a_frc_*, and *a_mix_* are the corresponding regression coefficients; *σ* is the Stefan-Boltzmann constant; *ψ* is the corresponding view factor; and *ε_sf_* and *ε_sr_* are the emissivity of the body surface and surrounding indoor space, respectively, The numeric phantom used is discretized by voxels; thus, its surface is approximately 1.4 times as large as that of an actual human [31]. The heat transfer coefficient is adjusted by the ratio between the actual and voxelized body surface area.

The heat transfer coefficient from skin to air was obtained from [30]. The specific heat, blood perfusion rate, heat conductivity and basal metabolism used herein are identical to those used in our previous study [32]. In addition, the blood perfusion rate through skin is the same as that used by McIntosh et al. [33]. The initial temperature distribution in a thermoneutral condition was determined using Equations (1) and (2) at ambient temperature *T_a_* = 30 °C, at which the thermoregulation measures, such as blood perfusion, sweating and blood temperature, remained constant.

#### 3.2.2. Blood Temperature Computation 

The blood temperature elevation was modeled so as to distinguish between arterial and venous temperatures, and thereby simulate for finer sites [28]. The blood temperature was changed according to [20].

(4) $$T_{bla,m}\left(t\right)=\frac{T_{blp}\left(t\right)\sum_{r}^{voxels}B\left(r,t\right)V\left(r\right)}{h_{x,m}+\sum_{r}^{voxels}B\left(r,t\right)V\left(r\right)}+\frac{h_{x,m}\sum_{r}^{voxels}T\left(r,t\right)B\left(r,t\right)V\left(r\right)}{\sum_{r}^{voxels}B\left(r,t\right)V\left(r\right)\left(h_{x,m}+\sum_{r}^{voxels}B\left(r,t\right)V\left(r\right)\right)},$$

(5) $$T_{blv,m}=\frac{\sum_{r}^{voxels}T\left(r,t\right)B\left(r,t\right)V\left(r\right)}{\sum_{r}^{voxels}B\left(r,t\right)V\left(r\right)},$$

where for each body part *m*, *T_bla,m_* and *T_blv,m_* denote the arterial and venous temperatures, respectively. The human model was divided into 13 parts (*m* = 13), as shown in Figure 3b [28], to compute the blood temperature in each body part. The blood temperatures of the head and torso were assumed to be the same based on the high blood volume [30]. The parameters *T_blp_*, *V*, and *h_x,m_* denote the central blood pool temperature, volume of tissues and coefficient corresponding to the counter-current heat exchange, respectively. The values of *h_x_* is determined based on the measured data [20].

#### 3.2.3. Modeling of Thermoregulatory Response

Several thermoregulatory response models have been proposed (see the review [13,34]). Among them, our algorithm is based on the model presented by Fiala et al. [28]. The blood perfusion rate through vasodilatation was changed in the same manner as in [16]. The evaporative heat loss on the skin is given as follows:

(6) $$\begin{array}{l} EV=\min\left\{SW(r,t)\cdot 40.6/S,\;EV_{\max}\right\},\\ EV_{\max}=2.2\cdot h_{c}f_{pcl}\left(P_{S}-\varphi_{\varepsilon }P_{A}\right),\\ h_{c}=3.0\sqrt{10v}, \end{array}$$

where *SW* is the sweating rate (see Equation (7)), *S* is the total surface area of the human body, and the factor of 40.6 is a conversion coefficient. The maximum evaporative heat loss *EV_max_* on the skin depends upon the ambient conditions. *h_c_* is the convective heat transfer coefficient as approximated by [35]; *v* is wind velocity; *P_S_* and *P_A_* are the saturated water vapor pressures at the temperature of the skin and at the ambient air temperature, respectively; *φ_e_* is the relative humidity of the ambient air; and *f_pcl_* is the permeation efficiency factor of clothing, which is affected by the speed of air movement. For simplicity, *f_pcl_* is assumed to be 1, corresponding to a naked body [36,37].

The sweating rate (*SW*) is assumed to depend on the temperature elevation in the skin and the hypothalamus, according to the following equation [30]:

(7) $$SW(r,t)=\gamma (r)\cdot \chi (r)\left[\begin{array}{l} \left\{\alpha_{11}\tanh\left(\beta_{11}\Delta T_{S}(t)-\beta_{10}\right)+\alpha_{10}\right\}\Delta T_{S}(t)\\ +\left\{\alpha_{21}\tanh\left(\beta_{21}\Delta T_{H}(t)-\beta_{20}\right)+\alpha_{20}\right\}\Delta T_{H}(t) \end{array}\right]+PI,$$

where ∆*T_S_* and ∆*T_H_* are the temperature elevations of the skin as averaged over the body and the hypothalamus temperatures, respectively. The insensible water loss (*PI*) is 0.71 g/min and 0.36 g/min for adults and children, respectively [16]. The multiplier *γ*(r) denotes the dependence of the *SW* on the body parts [38]. The coefficients *α* and *β* are estimated for an average *SW* based on the measurements in [35]. The *SW*s of the adult and child used here are the same as in the measurement literature [35].

As shown in Equation (7), the *SW* depends on the body temperature elevation, rather than the absolute temperature in the body. The measured *SW* of the child (integrated over the body) is comparable to that of an adult for a core temperature elevation of 0.5 °C [39]. A detailed explanation regarding the evaporative heat loss on skin was provided in our previous study, which considered the maximum evaporative heat loss depending on ambient conditions [36,37].

### 3.3. Electromagnetic Analysis

Both the solar (shortwave) and infrared (longwave) radiations were considered as incident radiations for the human models. The simulation of solar radiation was the same as in our previous studies [15,16]. The finite-difference time-domain (FDTD) method for Maxwell’s equation was used for calculating the power absorption in an anatomical human model. An in-house FDTD code, which was validated via inter-comparison, was used. The computational region was truncated by applying a twelve-layered, perfectly-matched layer absorbing boundary [36]. The dielectric properties of the tissues were determined with a Cole–Cole dispersion model [40]. For harmonically-varying fields, the power absorption was calculated from the Joule loss (*σE*^2^ in Equation (1)). 

For simulating the power absorption from solar radiation, the human models were exposed to a plane wave (uniform field) from the front, but considering the altitude. That is, the solar direction other than the altitude was not considered for clarity of power absorption in the following figures. If this direction was considered, the uncertainty of 3%–5% in core temperature was observed (not shown). The frequency was chosen as 6 GHz, at which the penetration depth of the radiative wave is 4 mm. Additional discussion regarding the validity of simulating incident solar radiation absorption can be found by Hanatani et al. [15]. 

A novelty here was the consideration of infrared (longwave) radiation flux calculated by the MSSG modeling, in which the three-dimensional radiative transfer processes, including scattering, were calculated based on the radiosity method. The infrared radiation was assumed to be evenly incident on the skin layer of the model. The absorption of the solar radiation was adjusted to consider the effects of clothing (short sleeve shirts and long pants). The infrared transmittance of the clothes was chosen as 0.32 [41].

## 4. Computational Results

The following scenario was used to demonstrate the differences in an adult and a child in a realistic environment. They walked from Nihon-bashi to Ginza, i.e., from north to south near the Tokyo station, along the street marked by blue in Figure 1d. A walking speed of 3.6 km/h (2.23 mph) was assumed for both the adult and child pedestrians for simplicity, although this is faster than the typical speed (~3 km/h) for a three-year-old child [42]. The metabolic equivalents to walking were estimated as 2.5 METs and 3.8 METs (where 1 MET is the metabolic equivalent of a sedentary person) for the adult and child, respectively [43,44]. The corresponding metabolic rate was uniformly added to the muscle over the body, i.e., considered as a whole-body exercise.

The adult and child were assumed to start walking at 14:00 JST from the north edge of the blue-marked street. It takes 33 min to walk through the street to the south edge at that assumed walking speed. We discarded the first 10 min to avoid the statistics being affected by the initial shock of the coupling of the weather and building-resolving simulations. The analysis therefore targeted the statistics for the 23 min while the adult and child were walking along the red-dashed line in Figure 1d.

Two routes were considered along the targeted street: one was the west side (shaded side) of the street, and the other was the east side (sunny side). Tall buildings faced the street, and they cast shadows (mostly on the west side). The street width was approximately 40 m, implying that the two routes were separated by approximately 8 grids in the 5 m-resolution building simulation.

### 4.1. Microscale Environment Simulation

**Figure 4:**
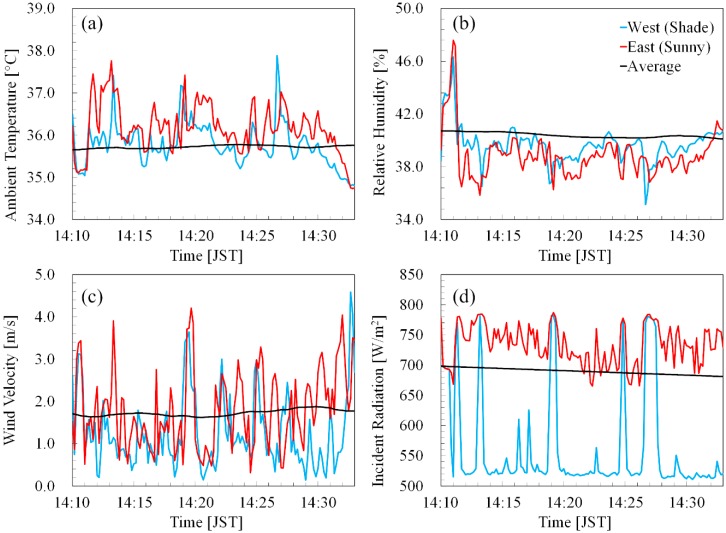
Time series of (**a**) ambient temperature, (**b**) relative humidity, (**c**) wind velocity and (**d**) incident radiation, which is the summation of the solar (shortwave) and infrared (longwave) incident radiations, at 2 m height in the pedestrian routes along the street indicated by the red-dotted line, part of the blue-marked line, in Figure 1d. One route took the shade-side (west side) of the street, and the other took the sunny-side (east side). The values from the shaded side are denoted by blue, and those from the sunny-side by red. The values averaged over the computational domain is denoted by black. A pedestrian walking speed of 1 m/s, i.e., 3.6 km/h, was assumed.

Figure 4 shows the time series of the atmospheric variables at a height of 2 m in the two pedestrian routes, along the street indicated by the red-dotted line in Figure 1d. The values obtained for the shade-side route are plotted in blue, and those for the sunny-side are plotted in red. The results averaged over the horizontal domain are also plotted for reference. The atmospheric temperature on the sunny side was, in general, higher than that on the shade side. The difference between the two routes was 1.5 °C at maximum, and 0.4 °C on average. The atmospheric variables in Figure 4 were considered in the computation of human thermal response.

In the present case, the whole-body-averaged power absorption per unit mass of the adult were 1.53 W/kg and 3.34 W/kg on the shaded and sunny sides, respectively, whereas those of the child were 2.64 W/kg and 5.81 W/kg on the sunny side and shaded side, respectively. They are comparable to those estimated from an incident power density of solar radiation (W/m^2^) multiplied by the body-surface-area-to-mass ratio.

### 4.2. Human Thermoregulation Simulation

**Figure 5:**
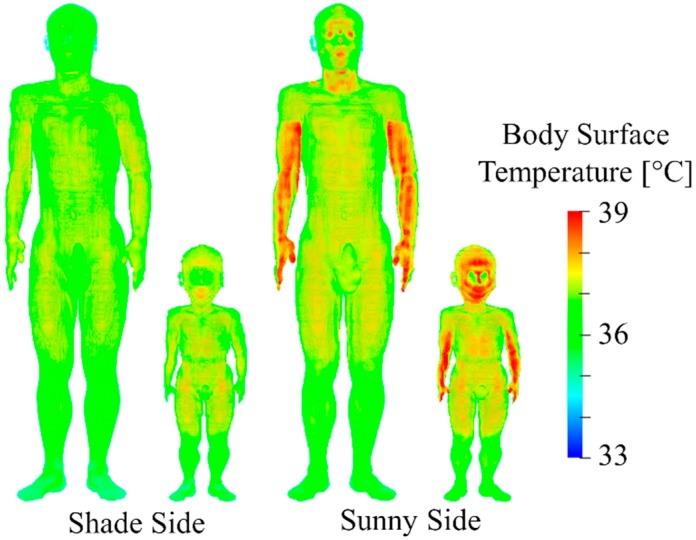
Distribution of surface temperature of adult and child for shade and sunny side. They were assumed to walk at 3.6 km/h for 23 min (14:10–14:33 JST on 7 August 2015).

Figure 5 shows the temperature distributions on the body surfaces of the adult and child models after 23 min of walking at 3.6 km/h (14:10–14:33 JST on 7 August 2015). As shown in Figure 5, the skin temperature for the models walking on the sunny-side was higher than that for models on the shade-side. The skin temperature elevations of the adult were 0.47 °C in the trunk and 1.11 °C in the face and arms for the sunny side, whereas they were 0.42 °C and 0.60 °C, respectively, for the shaded side. These temperature differences were more significant in the face and arms, which were directly exposed to sunlight. In the child model, the skin temperature elevations were 0.64 °C in the trunk and 1.97 °C in the face and arms for the sunny side, whereas they were 0.55 °C and 1.31 °C, respectively, for the shaded side. 

**Figure 6:**
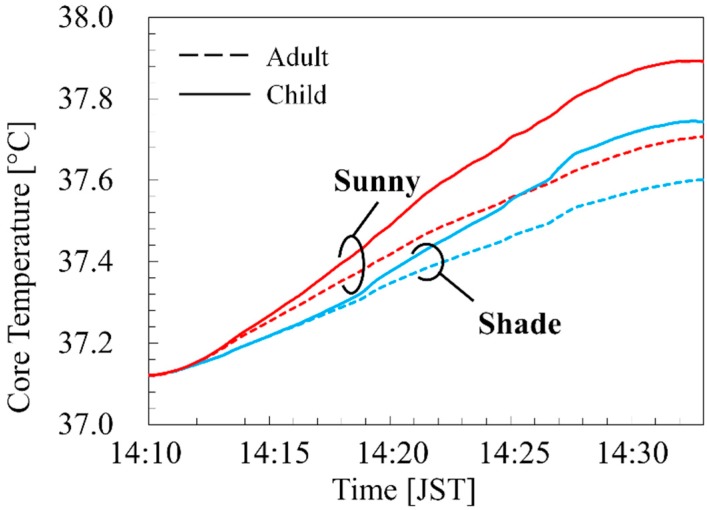
Time course of body core temperatures in adult and child walking at 3.6 km/h for 23 min on the route in Figure 1d.

Figure 6 shows the time course of the body core temperatures in the models of the adult and child. As shown in Figure 6, the core temperature of the adult elevated by 0.59 °C and 0.48 °C for 23 min on the sunny and shaded sides, respectively, whereas those of the child elevated by 0.77 °C and 0.62 °C on the sunny and shaded side, respectively. The differences in the temperature elevation between the sunny and shaded sides were slightly higher in the child than in the adult.

**Figure 7:**
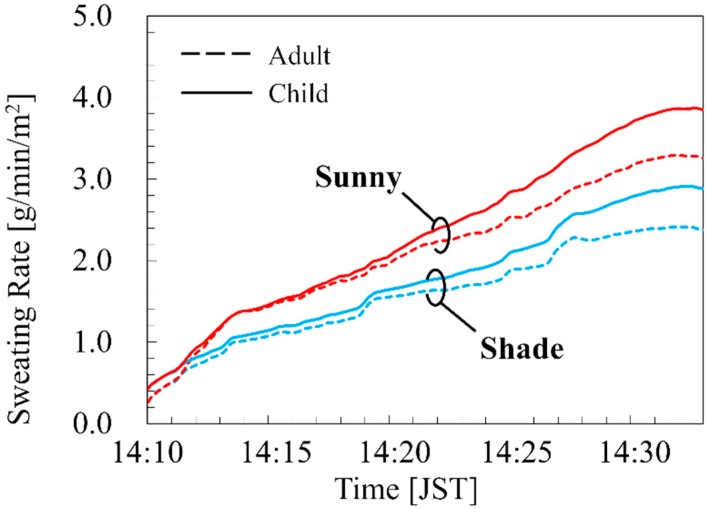
Sweating rates in adult and child walking at 3.6 km/h for 23 min on the route in Figure 1d.

Figure 7 shows the time course of the sweating per surface area in the adult and child models. As shown in Figure 7, the water loss of the adult reached to 3.25 g/min/m^2^ and 2.38 g/min/m^2^ for 23 min on the sunny and shaded sides, respectively, whereas those of the child reached to 3.85 g/min/m^2^ and 2.88 g/min/m^2^ on the sunny and shaded side, respectively.

**Figure 8:**
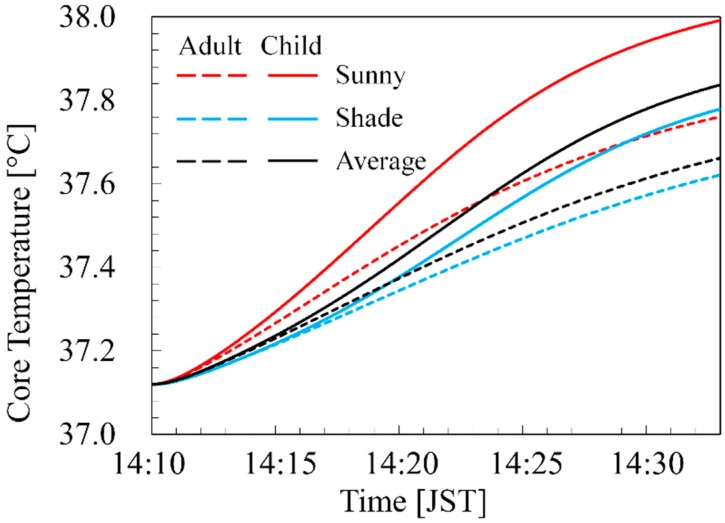
Time course of body core temperatures in adult and child walking at 3.6 km/h for 23 min with the constant ambient temperatures, 36.79 °C on the sunny side and 36.25 °C on the shaded side, on the route in Figure 1d. The black line shows the body core temperature with an ambient temperature and radiations averaged over the computational domain.

To demonstrate the effects of the time-course ambient conditions, the same scenarios were simulated with a constant ambient temperature, i.e., one scenario is the domain-averaged thermal environment and the other is 36.79 °C on the sunny side and 36.25 °C on the shaded side, at 14:20 JST on 7 August 2015. Figure 8 shows the time course of the body core temperatures in the adult and child in the constant ambient temperatures for 23 min. As shown in Figure 8, the core temperature elevation with the domain-averaged thermal environment were 0.54 °C in the adult and 0.72 °C in the child. In addition, the difference in the body core temperature elevation between the sunny and shaded sides was 0.14 °C in the adult and 0.21 °C in the child.

## 5. Discussion

In this study, we presented the temperature elevation and sweating in an adult and child exposed to a highly accurate environment computed by a building-resolving LES of a microscale thermal environment, while considering the coupling of the wind and radiative fields. The thermoregulation model used here has been validated in chamber as well as comparing with previous studies in different conditions [16,19,20]. With the integration of two different computational methods, we then, for the first time, demonstrated the differences in body core temperature elevation owing to differences in age, in addition to a spatiotemporal variation of the ambient conditions including mean radiant exposure.

### 5.1. Atmospheric Temperature

As shown in Figure 4, the domain-averaged values did not change much over time, whereas the values for the two pedestrian routes did change significantly. In terms of the incident radiation, the domain-averaged results were similar to the results for the sunny side route, because the sunny area was larger than the shaded area in the targeted period. 

Interestingly, the temperature shows the opposite tendency: The domain-averaged results were more similar to the results for the shaded side route. This can be explained by the instability induced by locally high temperature. The air with locally high temperature is light and buoyant, leading to convection and mixing. Consequently, the area with locally high temperature tends to be limited. The observed relatively low temperature and high humidity at the beginning were brought by the cool and humid air from the river.

### 5.2. Comparison of Temperature Elevation and Water Loss for the Sunny and Shaded Sides in Adult and Child

As shown in Figure 5, Figure 6 and Figure 7 the skin and core temperature elevations and water losses on the sunny side were higher than that in the shade. The differences in the core temperature elevations of the adult and child between the sunny and shaded sides were 0.11 °C and 0.15 °C, respectively, as compared to the approximately 0.4 °C difference in the averaged ambient temperature [45]. The amounts of the water loss to weight ratio of the adult were 0.13% and 0.10% on the sunny and shaded sides, respectively, whereas those of the child were to 0.23% and 0.18% on the sunny and shaded side, respectively. The differences in the amounts of water loss to weight ratio between the sunny and shaded sides were slightly higher in the child than in the adult. This result suggested that selecting a suitable walking route can manage the risk of heat-related illness. In this computation, the contribution of long- and shortwaves to the temperature elevation was 77% in the shaded and 82% in the sunny side, respectively, suggesting the importance of the detail consideration of incident radiations.

As shown in Figure 5 and Figure 6, the skin and core temperature elevations of the child were higher than those of the adult. These results suggest that the difference in the risk of heat-related illness depends on the difference in age from a physical viewpoint. This is primarily attributed to the difference in the surface area-to-mass ratio between the adult and child [46]. A higher ratio confers a greater capacity to exchange heat with the ambient temperature. In addition, the power absorption from solar radiation is greater due to the ratios. The ratios of surface area-to-mass were 0.027 m^2^/kg and 0.043 m^2^/kg in the adult and child, respectively [27]. However, the difference of the core temperature elevation is 131%, and is not proportional to that in the area-to-mass ratios (159%). This is also classified into two factors: ambient heat and radiation. The former should simply be proportional to the body-surface-area to mass ratio. Instead, the latter may be inversely proportional to this. Radiation absorption is approximately proportional to the surface area, but cooling is also proportional to it. Thus, we found that in a realistic environment it is not straightforward to estimate without detailed computation.

Comparing Figure 6 and Figure 8, it can be seen that the body core temperature elevations were different even during the 1.4 km walking, considering the spatiotemporal variation of the ambient environment. These differences could increase with longer walking distances. According to the JMA data, the ambient temperature at the relevant time was 35.9–36.5 °C.

The ACGIH [5] defines a 1 °C body core temperature elevation as the limit for healthy workers in an occupational environment. The body core temperature of the child for the sunny side did elevate by 0.87 °C under this situation for 23 min, and then remained at a steady state of 1.01 °C at 60 min. The water losses of the adult and child when in the sunny side for 60 min were 402.0 g and 142.0 g, respectively. They were approximately 0.66% and 1.09% of the body weight of the adult and child, respectively. This result shows that the risk of dehydration in children is higher than that in adults.

Thermal comfort is closely related to the skin temperature [13,47]. In addition, the skin temperature was an important indicator of the control of human thermoregulatory [48]. The skin temperature distribution obtained by the developed techniques may be applied, such as real-time thermal comfort assessment. Further, these techniques may be useful for caregivers in selecting actions to mitigate the risk of heat-related illnesses in children.

### 5.3. Numerical Uncertainly and Limitation

The numerical uncertainly in this study can be summarized as follows. First, for simplicity, we used the ambient temperature at a 2 m height, i.e., the same height for both adult and child. However, the ambient temperature depends upon the height in the actual environment. One of the factors that increases the risk of heat-related illness in children is that they are exposed to higher ambient temperatures than adults, because they are close to the ground. However, this factor is not easy to quantify, as no exact measurement has been reported. Thus, it remains a topic for future study. 

As mentioned in Section 3.2, we selected the thermoregulation of the human models of an adult grown in a temperate zone [20]. Core temperature elevation and water loss for people who grow in the tropical or frigid zone may differ. The uncertainty of thermophysiological parameters was discussed in our previous studies [37,49]. The computed core temperature may be different, 10%–20%, depending on the value of blood perfusion and the heat transfer to ambient temperature.

The uncertainty in the thermoregulation of children is worth mentioning, as no measured data has been reported for core temperature elevations higher than 0.5−0.6 °C. The computational modeling would be close, but somewhat higher than this range, though, and may still provide a good approximation. Finally, we assumed that the transmittance of the clothes was 0.32, based on white cotton [41]. Several literatures have reported that the effects of clothing on skin temperature are caused by light penetration in the fabrics, which depends on many factors, such as the colors and materials of the clothing [41,50]. For example, the heat absorption owing to sunlight in black garments is greater than that in white garments. Thus, children wearing a clothing with dark color may experience a higher core/skin temperature elevation. Computed core temperature elevation was a 37% increase in the model without any clothing.

In this study, we did not present the data for the elderly, because they suffer from heat-related illness mainly in their home rather than the outside [51]. Note that we developed the thermoregulatory model for the elderly in Hirata et al. [18]. We computed the core temperature elevations of elderly in the same scenario of Figure 5, and were 13% higher than those of the adult.

In general, the risk of heat-related illness also varies depending individual factors, such as accommodation, motion intensity and chronic diseases. The computational model [16,18,20] integrated here may be able to apply to the population, except for those with chronic disease. This is because the computed model considering chronic diseases has not yet been developed. The same approach also may not be directly applied to those with chronic disease, because other factors may be considered. Note that as is similar to the elderly, in general, people with such chronic disease may not be active in a hot environment.

## 6. Conclusions

We developed a methodology to estimate the time-course core temperature and water loss on street walkers in different age groups. Urban micrometeorology prediction with a high spatiotemporal resolution was utilized to reproduce the environment surrounding the walkers. Specifically, we performed a building-resolving LES, coupled with a three-dimensional radiative transfer model, with a 5-m spatial resolution for a 2 km × 2 km horizontal domain in the center of Tokyo, Japan. We then extracted the time series of atmospheric variables, such as the ambient temperature and radiative heat fluxes for pedestrians on an actual walking route. Finally, we demonstrated the temperature elevation in the human bodies, considering the time course of ambient temperature, humidity, wind velocity and heat radiation. The difference attributable to urban micrometeorology and human morphology was notable. 

The present techniques are the fundamental technology for the behavior selection to manage the risk of heat-related illness. In the future work, the present integrated technology can be coupled with a multi-agent model to account for individual behavior, so as to assist in the selection of measures to offset the risk.
